# Percutaneous stenting of left hepatic vein followed by Ex vivo Liver Resection and Autotransplantation in a patient with hepatic alveolar echinococcosis with Budd-Chiari syndrome

**DOI:** 10.1016/j.ijscr.2020.03.004

**Published:** 2020-03-09

**Authors:** Yu Zhang, Ping Xie, Chong Yang, Hongji Yang, Jun Liu, Guo Zhou, Shaoping Deng, Wan Yee Lau

**Affiliations:** a Organ Transplantation Center, Sichuan Academy of Medical Sciences & Sichuan Provincial People’s Hospital, School of Clinical Medicine, University of Electronic Science and Technology of China, Chengdu 610072, Sichuan, China; b Ultrasonography Department, Sichuan Academy of Medical Sciences & Sichuan Provincial People’s Hospital, School of Clinical Medicine, University of Electronic Science and Technology of China, Chengdu 610072, Sichuan, China; c Faculty of Medicine, The Chinese University of Hong Kong, Prince of Wales Hospital, Shatin, N.T., Hong Kong Special Administrative Region

**Keywords:** Hepatic alveolar echinococcosis, Budd-Chiari syndrome, Hepatic venous stenting, Autotransplantation

## Abstract

**Background and aims:**

Infiltration of hepatic venous outflow in hepatic alveolar echinococcosis can lead to development of Budd-Chiari syndrome. Medical treatment of this condition is generally unsatisfactory. Radical hepatic resection is impossible for extensive parasitic involvement of liver. This is a case report on a patient who was successfully treated with percutaneous stenting of left hepatic vein followed by Ex vivo Liver Resection and Autotransplantation (ELRA).

**Methods:**

Using the transjugular approach, a metal mesh stent was placed in the left hepatic vein of a 45-year-old man who presented with Budd-Chiari syndrome. After disappearance of ascites and improvement in liver function, Ex vivo Liver Resection and Autotransplantation were performed.

**Results:**

The patient underwent left hepatic vein stenting for Budd-Chiari syndrome without complication. Three months later, liver biopsy showed fibrous proliferation of interlobular portal areas and normal hepatic lobules. After successful Ex vivo Liver Resection and Autotransplantation, follow-up examination at 6 months showed normal liver function and no evidence of recurrence.

**Conclusions:**

Allotransplantation is an accepted treatment for advanced hepatic alveolar echinococcosis with Budd-Chiari syndrome. However, shortage of organ donors and need for immunosuppression are challenging problems. In selected patients with hepatic vein stenoses presenting as Budd-Chiari syndreme, percutaneous stenting of hepatic veins followed by ex vivo liver resection and autotransplantation represent an alternative curative treatment option.

## Highlights

• A metal mesh stent was placed in the left hepatic vein of a 45-year-old man who presented with Budd-Chiari syndrome in stage 1.
• After disappearance of ascites and improvement in liver function, ELRA was performed in stage 2.
• Follow-up examination at 6 months showed normal liver function and no evidence of recurrence.
• In selected patients with Budd-Chiari syndreme, percutaneous stenting followed by ELRA represent an curative treatment option.

## 1 Introduction

Hepatic alveolar echinococcosis (HAE) is a life-threatening parasitic disease caused by the larvae, Echinococcus multilocularis. It behaves like a malignant tumor and often leads to comprehensive invasion of multiple intrahepatic structures [1].

Infiltration of hepatic veins in HAE can lead to the development of Budd-Chiari syndrome. Portal hypertension with ascites is a common presentation. Although medical therapy may stabilize the disease for some time, long-term outcome is generally unsatisfactory. Percutaneous stenting of hepatic veins represents a promising treatment option [2], but definitive cure can only be obtained by resection of the localized liver masses. Most lesions are impossible or difficult to resect in an advanced stage, being characterized by extensive infiltration of intrahepatic structures [3].

Liver transplantation is an accepted treatment for patients with HAE for whom there are no other medical or surgical treatment options [4,5]. However, liver transplantation for these patients remains highly controversial. The need for an organ donor followed by life-long immunosuppression with an increased recurrence risk are the reasons put forward against allotransplantation. Even for those clinicians who support allotransplantation, the decision to proceed to transplantation “should be cautiously considered” [6]. Ex vivo liver resection and autotransplantation (ELRA) was firstly introduced by Pichlmayr in 1988 to treat conventionally unresectable malignant tumors confined to the liver [7]. ELRA is feasible to treat end-stage HAE that does not require an organ donor or immunosuppressive therapy [[8], [9], [10], [11]]. However, precise preoperative assessment and strict patient selection are of utmost importance [12]. Most patients presenting with Budd-Chiari syndrome need allotransplantation because of poor clinical conditions with ascites and jaundice [[13], [14], [15]]. For end-stage HAE presenting with Budd-Chiari syndrome, ELRA is unfeasible due to the bad “quality” of the future remnant liver (FLR) [12].

This is a case report on a patient who underwent percutaneous stenting of left hepatic vein to achieve improvement in “quality” of the FLR and disappearance of ascites, followed 3 months later by ex vivo liver resection and autotransplantation (ELRA) to treat Budd-Chiari syndrome. To our knowledge, this is the first case report. This work has been reported in line with the SCARE criteria [16].

## 2 Case report

**Fig. 1:**
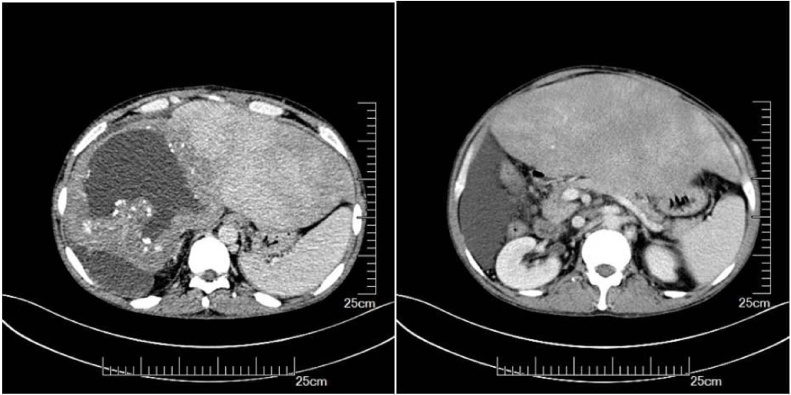
Delayed uptake of contrast medium in the hepatic parenchyma and massive ascites.

**Fig. 2:**
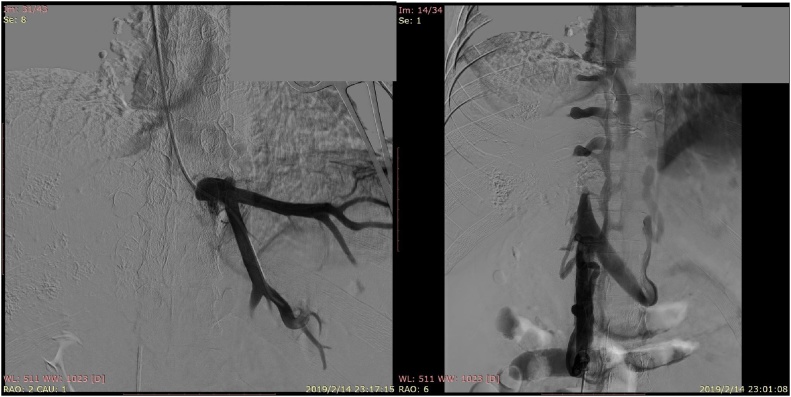
a and b The stenoses of the left hepatic vein was confirmed by transjugular phlebography and retrohepatic segment of inferior vena cava were completely occluded.

A 45-year-old man with advanced stage HAE was transferred to our center from Qinghai Province, a pasturing area in Western China. Two years ago, the patient was diagnosed to suffer from HAE by enhanced computerized tomography (CT). An indirect echinococcal hemagglutination test, and an anti-E granulosus IgG test were both positive. He refused to undergo liver resection and was started on albendazole (400 mg) 3 times daily. The liver lesion continued to grow infiltratively. The patient’s condition worsened, and he developed massive ascites. CT scan depicted the lesion to be in the right liver, with invasion to the inferior vena cava, middle and right hepatic veins, and stenosis of left hepatic vein. There was also involvement of the porta hepatis and splenomegaly. Fig. 1 shows significant delay in uptake of contrast in the hepatic parenchyma with severe congestion, indicating significant venous outflow obstruction of the liver. Endoscopy disclosed grade II oesophageal varices extending to gastric fundus. Stenoses of left hepatic vein was subsequently confirmed by transjugular phlebography of hepatic veins and verified by measurement of the prestenotic to poststenotic venous pressure with a difference of 54.3 cmH_2_o (Fig. 2a). The middle and right hepatic veins and retrohepatic segment of inferior vena cava were completely occluded (Fig. 2b). The pressure of the poststenotic inferior vena cava was 36.6 cmH_2_o. Liver function was significantly compromised.

**Fig. 3:**
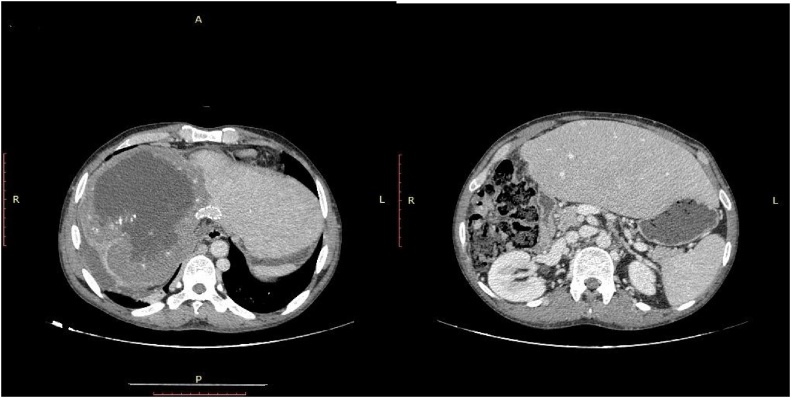
a and b The transjugular placement of self expanding metal mesh stents and congestion of liver was disappeared.

Both the ascites which had persisted for more than three months, and the compromised liver function were indications for transjugular intrahepatic portosystemic shunting. Instead, with angiographic evidence of left hepatic vein stenosis with a high pressure gradient, transjugular placement of a self-expanding metal mesh stent (William Cook Europe Aps, Sandet 6,DK-4632 8/60 mm) in the left hepatic vein (Fig. 3A) was decided. Immediately after stenting, the stenosis of the left hepatic vein disappeared and left liver venous pressure difference decreased to 31.2 cmH_2_o.

At a follow-up examination one week later, ultrasound showed only remaining discrete ascites. At a follow-up of 3 months, the patient felt subjectively well. Both Doppler ultrasound and enhanced CT continued to show stent patency and there was no discernible ascites. Endoscopy showed no oesophageal varices. Liver function improved significantly. The ICG R15 was 6.7%. Liver biopsy showed fibrous proliferation of interlobular portal areas and normal hepatic lobules. His body weight was 60 kg. The standard liver volume based on the West China formula was 1018 mL [17]. CT indicated that congestion of liver disappeared and the volume of the future remnant liver was 1180 mL and the predictive graft-to-recipient weight ratio (GRWR) was 1.97% (Fig. 3b).

The surgical procedure and this report were approved by the Ethics Committee of Sichuan Provincial People's Hospital.

## 3 Operation procedure

A Mercedes Benz incision was used. After mobilization of the liver from the adjacent adhesions, the portal vein (PV) pressure as measured by intubation of the right gastrioepiploicgastroepiploic vein was 25.8 cmH2o. The porta hepatis was dissected to isolate the PV, hepatic artery (HA) and common bile duct. The hepatic veins and the IVC were then dissected. The lesion was found to invade the roots of the hepatic veins, portal veins and bile ducts. The suprahepatic and subhepatic IVC were dissected. The liver parenchyma was split along the right border of the falciform ligament using the anterior approach. The left branch of PV was invaded. The root of the left HV was exposed to show the invasion by the lesion for about 3 cm in length. The right HV, middle HV and retrohepatic IVC were all invaded. It was impossible to reconstruct the blood vessels in situ, so the specimen was taken out from the patient and was moved into an ice bath for bench surgical resection and blood vessel reconstruction.

**Fig. 4:**
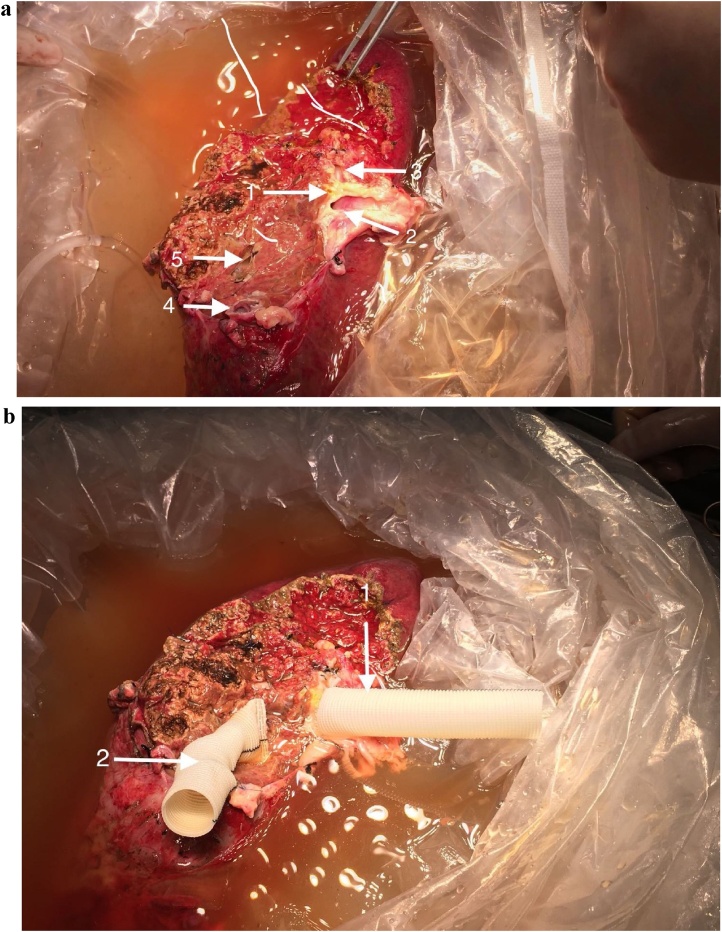
a Ex vivo liver resection was performed for the liver AE lesion. 1: left hepatic biliary duct opening. 2: PV opening of segment II. 3: PV opening of segment III. 4: HV opening of segment II. 5: HV opening of segment III. b The left PV and outflow of the left HV to the IVC was reconstructed using artificial blood vessel for a wide mouth anastomosis. 1: left PV. 2: left HV.

The liver was perfused with 3000 mL of 4 °C University of Wisconsin (U-W) solution via the PV branch, and the HA and bile duct were simultaneously perfused with 200 mL of U-W solution using syringe injection. Ex vivo liver resection was performed for the HAE lesion. Parenchymal liver transection was performed with a minimum of 1.0-cm lesion-free margin (Fig. 4a). The autograft was inspected carefully by repeated perfusion to detect any bile leakage or vascular defects. The left PV and outflow of the left HV to the IVC waswere reconstructed using artificial blood vessels (Fig. 4b).

**Fig. 5:**
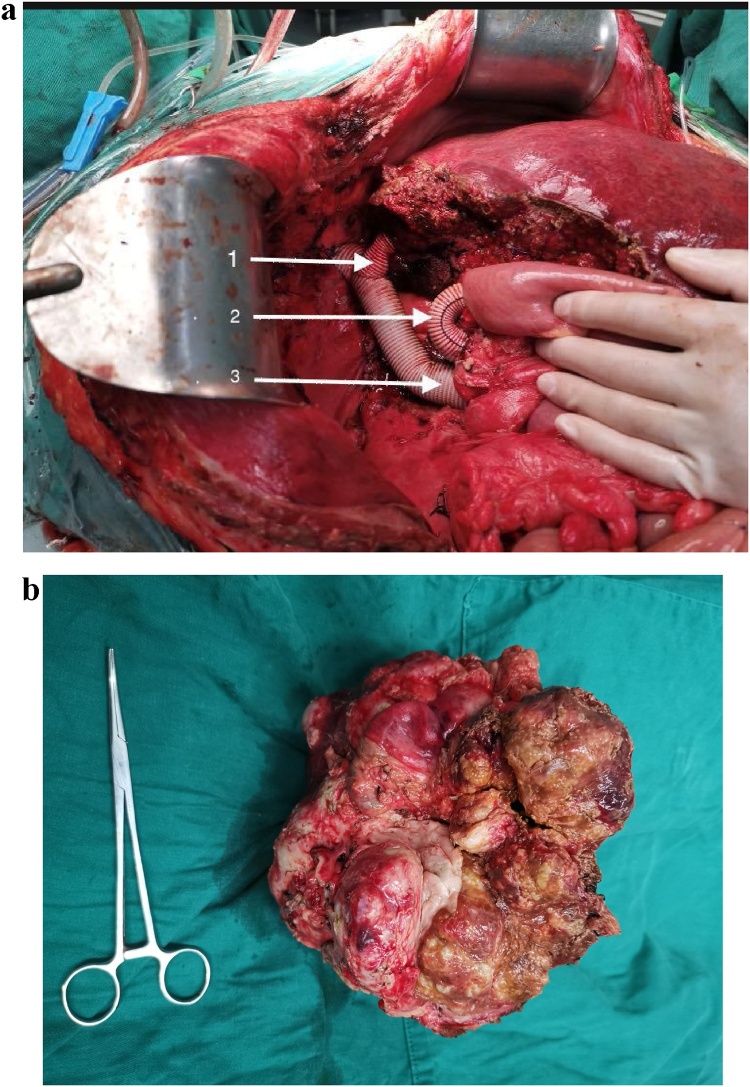
a The left HV was reconstructed to IVC using end-to-side anastomosis. The left-PV and left-HA were reconstructed using end-to-end anastomosis. The left-HB was reconstructed using bilioenterostomy. 1: left HV, 2: left-PV, 3: IVC. b The resected specimen.

The left HV was anastomosed to IVC with a wide-mouth anastomosis using an end-to-side anastomosis. The left-PV and left-HA were reconstructed using an end-to-end anastomosis, respectively. The left hepatic duct was reconstructed using bilioenterostomy (Fig. 5a). At the end of the operation, the measured PV pressure was 21cmH_2_o. The resected specimen was sent for histopathological examination (Fig. 5b).

## 4 Postoperative therapy

**Fig. 6:**
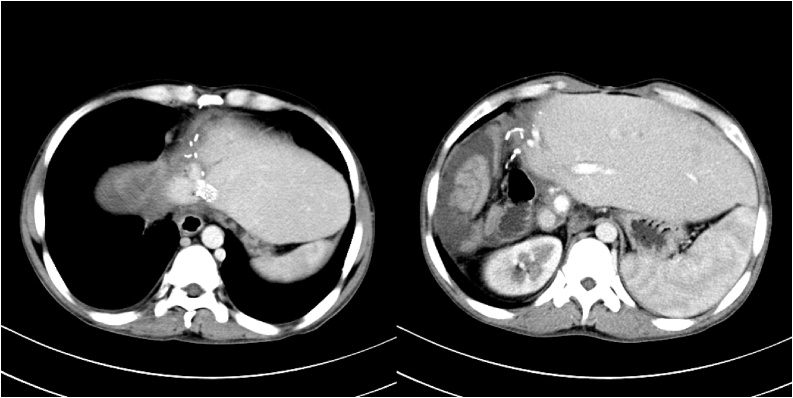
The CT scan indicated no recurrence, thrombus, liver congestion and cholangiectasis.

The surgical procedure lasted for 10 h, and blood loss was 800 mL. The anhepatic phase lasted for 2 h. This patient was admitted to the Intensive Care Unit (ICU) after surgery. A low-molecular-weight heparin sodium injection (2050 IU, 12 h) was started after the patient was stable hemodynamically. The dosage was adjusted according to the weight of the patient and the international normalized ratio (INR). When the patient began eating, the low-molecular-weight heparin sodium was discontinued and changed to warfarin sodium tablets. The individual dosage was adjusted according to the weight and the INR of the patient with a reference of 2.0–3.0. This patient was transferred from the ICU to the general ward 3 days after operation. He did not develop any complications. The histopathological examination confirmed HAE lesion. CT scan 6 months post-operation revealed there was no thrombosis in the reconstructed left PV, left HA, left HV and IVC. The CT scan indicated no hepatic nodules or local recurrence. There was no liver congestion, cholangiectasis and stenosis of the duct-jejunum anastomosis (Fig. 6).

## 5 Discussion

Currently, radical resection combined with albendazole therapy is the treatment of choice for patients with HAE [1,2]. However, intensive and complex involvement of intrahepatic vessels and bile ducts in patients with end-stage HAE can present with difficulties to radical resection. Liver transplantation has provided an alternative for patients with “unresectable’’ but not metastatic HAE lesions. Despite relatively good long-term survival when compared with liver transplantation for cancer, the relatively high mortality rate within the first year of transplantation, shortage of liver donors, and mandatory use of immunosuppressive agents leading to postoperative relapse have limited the clinical application of allogeneic liver transplantation in HAE patients [18,19]. To overcome these problems, ex vivo liver resection and autologous liver transplantation (ERAT) using living donor liver transplantation technique have been used to treat patients with end-stage HAE [[8], [9], [10], [11]].

Interventional methods (stent placement, angioplasty, thrombolysis) are often use to treat patients presenting with Budd-Chiari syndrome. The appearance of ascites with clinical evidence of portal hypertension led us to defer surgical treatment. Interventional treatment with percutaneous stenting of the left hepatic vein was first carried out. After disappearance of ascites and improvement of “quality” of functional liver, ex vivo liver resection and autotransplantation was performed as a radical treatment. To our knowledge, our case report was the first to use hepatic vein stenting followed by ELRA to treat HAE. In the 6 month follow-up, the liver function test was normalized and there was no evidence of thrombosis and HAE relapse.

In conclusion, in selected patients with advanced HAE presenting with Budd-Chiari syndrome, percutaneous stenting of hepatic vein followed by ex vivo liver resection and autotransplantation represents an alternative curative procedure.

## Declaration of Competing Interest

No any conflicts of interest.

## Sources of funding

This work was supported by grants from Chendu branch, Chinese Academy of Sciences, Health Department of Sichuan Province, China (No. 150192) and Funding of Sichuan Academy of Medical Sciences.

## Ethical approval

This case was approved by the ethical committee of the Sichuan Academy of Medical Sciences (Sichuan Provincial People’s Hospital), and it followed the ethical guidelines of the 1975 Declaration of Helsinki.

## Consent

Written informed consent was obtained from the patient for publication of this case report.

## Author’s contribution

Wan Yee Lau and Shaoping Deng have contributed equally to this work; Yu Zhang and Ping Xie have contributed equally to this work. Wan Yee Lau, Shaoping Deng, Yu Zhang and Pingxie designed the study; Chong Yang, Hongji Yang, Jun Liu and Guo Zhou collected the patient’s clinical data; Yu Zhang and Wan Yee Lau analyzed the data and wrote the paper.

## Registration of research studies

Researchregistry5206.

## Guarantor

Yu Zhang, Wan Yee Lau.

## Provenance and peer review

Not commissioned, externally peer-reviewed.
